# A Molecular Dynamics Study of Crosslinked Phthalonitrile Polymers: The Effect of Crosslink Density on Thermomechanical and Dielectric Properties

**DOI:** 10.3390/polym10010064

**Published:** 2018-01-11

**Authors:** Janel Chua, Qingsong Tu

**Affiliations:** 1 DSO National Laboratories, Singapore 118225, Singapore; csonglin@dso.org.sg; 2 Department of Civil and Environmental Engineering, University of California, Berkeley, CA 94720, USA

**Keywords:** phthalonitrile polymer, cross-linking density, molecular dynamics, thermal expansion, dielectric constant

## Abstract

In this work, molecular dynamics (MD) and molecular mechanics (MM) simulations are used to study well-equilibrated models of 4,4′-bis(3,4-dicyanophenoxy)biphenyl (BPh)–1,3-bis(3-aminophenoxy)benzene (m-APB) phthalonitrile (PN) system with a range of crosslink densities. A cross-linking technique is introduced to build a series of systems with different crosslink densities; several key properties of this material, including thermal expansion, mechanical properties and dielectric properties are studied and compared with experimental results. It is found that the coefficient of linear thermal expansion predicted by the model is in good agreement with experimental results and indicative of the good thermal stability of the PN polymeric system. The simulation also shows that this polymer has excellent mechanical property, whose strength increases with increasing crosslink density. Lastly and most importantly, the calculated dielectric constant—which shows that this polymer is an excellent insulating material—indicates that there is an inverse relation between cross-linking density and dielectric constant. The trend gave rise to an empirical quadratic function which can be used to predict the limits of attainable dielectric constant for highly crosslinked polymer systems. The current computational work provides strong evidence that this polymer is a promising material for aerospace applications and offers guidance for experimental studies of the effect of cross-linking density on the thermal, mechanical and dielectric properties of the material.

## 1. Introduction

In the past 50 years, a considerable amount of research has been conducted on high temperature polymers for aerospace applications. For a long time, most high temperature polymer composite materials were based on polymeric systems which are functional up to ~400 °C. Applications that required an operating temperature of >400 °C had to rely on denser high temperature materials such as ceramic systems or metallic alloys. A breakthrough came, however, when Keller from Naval Research Laboratory [1] started developing thermosetting phthalonitriles for potential aerospace, marine and electronic packaging applications at temperatures >400 °C. Unlike most of its thermoset predecessors which cure by a process that creates a lot of voids due to release of volatile by-products, phthalonitrile polymer systems are formed via the addition cure mechanism where phthalonitrile based monomers react with aromatic amines such as 1,3-bis(3-aminophenoxy)benzene (m-APB) or bis[4(4-aminophenoxy)phenyl]sulfone (p-BAPS) to produce a highly-crosslinked system. This curing method ensures that little to no volatiles are evolved during polymerization leading to highly crosslinked, void-free polymer networks that have good mechanical properties: with almost the same specific compressive strength as concrete and only 6% the density of concrete when in its foamed form [2,3]. Besides its exceptional mechanical properties, this material also shows outstanding thermo-oxidative stability at elevated temperatures and superior fire resistance [1]. In addition, there has been an increased interest in studying the electrical properties of phthalonitrile with fillers (such as graphene flakes or silver nano-particles) to explore its potential application in microscale composite electrical devices where high mechanical strength, low density and good electrical properties are of interest [4]. In order to investigate the aforementioned properties of the PN polymer system, directed trial and error approaches could be employed to optimize the experimental processing conditions. Such methods are however potentially time-consuming and expensive. In order to save on natural and human resources, researchers have turned to computer simulations as a means to understanding these polymer systems. On one hand, they can reduce trial and error experimentation; on the other hand, they provide insights at the molecular and micro length scales which are often difficult to obtain from experiments.

One of the polymer systems that were used as a pioneering test case in molecular modelling of polymers was the epoxy system [5]. Over the last 20 years, MD simulations based on bead-spring models and Monte-Carlo simulations based on bond-fluctuation model have been used to study the molecular behavior of polymeric materials [6,7]. Most of these studies were however primarily mathematical in nature and did not take into account the topological information on the network. As a result of this, useful information related to mechanical as well as thermodynamic properties was not accounted for. Soon after, several authors studied the formation of crosslinked epoxy with topological information using different approaches of simulated cross-linking. Doherty et al. [8] created polymethacrylate (PMA) networks using lattice-based simulations using the polymerization molecular dynamics scheme. Yarovsky et al. [5] discussed a methodology to cross-link low molecular weight water soluble phosphate-modified epoxy resin (CYMEL 1158). However, all the cross-linking reactions were carried out simultaneously (static cross-linking) in a three-step procedure in their study. Cross-linking of epoxy resins using molecular dynamics was performed by Xu et al. [9] and their model was used to study diffusion of water in epoxy’s crosslinked networks. The authors used an iterative Molecular Dynamics (MD)/Molecular Minimization (MM) procedure to crosslink an epoxy resin where the newly formed topology was subjected to 1000 MD steps of relaxation, with one crosslink established per iteration. Other computational studies involving epoxies [10,11,12] have been conducted.

Although the aforementioned studies have made significant progress in the molecular modelling of crosslinked epoxies, similar models have not been developed for additional cured polymeric systems that contain multiple cross-linking steps and dielectric properties of crosslinked polymeric systems have also not been widely studied at the molecular length scale. Thus, in the spirit of the above discussion, a molecular dynamics study on a crosslinked phthalonitrile polymeric system was conducted. In the paper, we examined PN polymer, a thermally stable resin synthesized by Keller from USA Naval Research Laboratory [1] which was later successfully made into a carbonized foam by Liying Zhang et al. from Nanyang Technological University, Singapore [2]. An efficient method of creating a molecular model of crosslinked phthalonitrile system containing ~10,000 atoms is described. Three models were built with different percentages of crosslink and the model predicts that the thermal expansion coefficient and elastic properties are dependent on crosslink density. Due to the potential electromagnetic interference (EMI) properties of carbonized phthalonitrile polymer [2], dielectric properties were also obtained for pre-carbonized crosslinked resin to serve as a reference point for future carbonization studies.

The paper is organized as follows. In Section 2, the molecular modelling is presented with the main focus on cross-linking procedure and systems with different cross-linking densities are modelled and discussed. In Section 3, material properties including thermodynamic, elastic and dielectric are presented as a function of crosslink density. Due to specific interest in the superior thermal and mechanical properties of phthalonitrile polymeric systems, the first two parts look at MD simulated thermal and mechanical properties. In the third part, the dielectric properties of pre-carbonized phthalonitrile polymers are studied in order to observe the change in dielectric constant as a function of carbonization in future work. A method for obtaining the dielectric constant of polymers using MD was established which will be applied to future work on carbonized phthalonitrile polymeric systems.

## 2. Material and Molecular Modelling

### 2.1. Simulation Details

**Figure 1:**
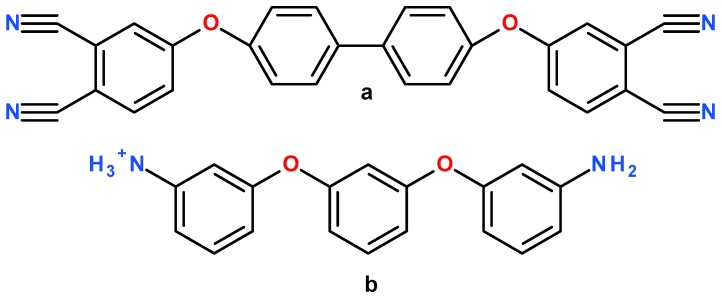
Molecular structure of (**a**) BPh monomer and (**b**) activated m-APB cross-linking agent.

The PN resin system modelled in this paper consists of the 4,4′-bis(3,4-dicyanophenoxy)biphenyl (BPh) monomer and the cross-linking agent 1,3-bis(3-aminophenoxy)benzene (m-APB). The molecular structure of BPh and activated m-APB are shown in Figure 1.

**Figure 2:**
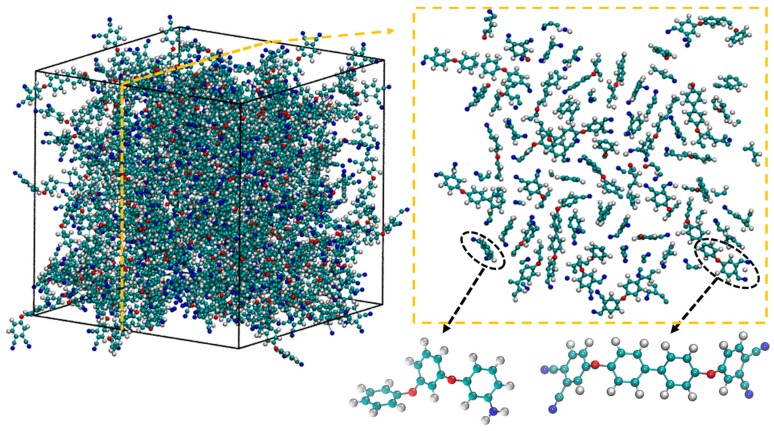
Initial uncrosslinked model of BPh–substitute m-APB mixture with ratio 28:1 (“**red**” represents Oxygen, “**white**” is Hydrogen, “**blue**” is Nitrogen and “**green**” is Carbon); 3D structure is depicted on the left and cross-section of the middle is depicted on the right.

The initial uncrosslinked molecular model structure was established using a procedure similar to that used by Varshney et al. [12]. In accordance with experimental measurements conducted by Keller et al. [13] a molar ratio of 28:1 (BPh:m-APB) with a density of 1.2 g/cm^3^ is selected in this study. Image of the initial uncrosslinked structure along with a 3D molecular structure of the cross-linking agent and monomer are shown in Figure 2 below. The 3D structure is shown in the left and a cross section of the middle is shown on the right, curing agents are initially homogeneously distributed without any cross-linking. A homogeneous distribution was selected as it most closely mimics macroscale experimental conditions where we may assume that the curing agents are somewhat evenly distributed in the polymeric system. Should the distribution of curing agents be inhomogeneous, the effect in real life experiments and in modelled systems would be lower levels of cross-linking at saturation. This is due to the uneven cross-linking at the initial stages of cross-linking which results in steric hindrance at later stages of cross-linking. In our current study, we consider the case of a homogeneously distributed initial configuration in order to maximize the cross-linking levels at saturation.

**Figure 3:**
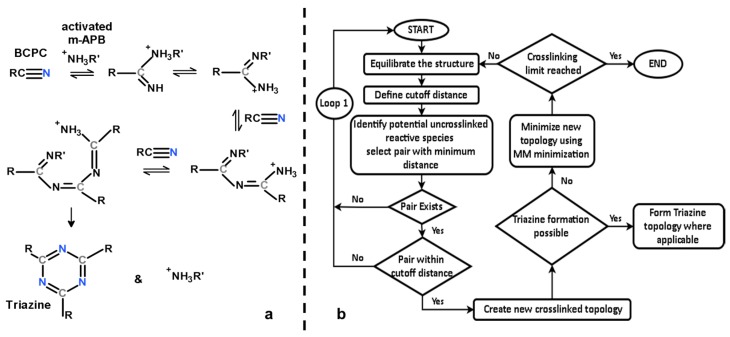
(**a**) Cross-linking reactions leading up to triazine formation; (**b**) cross-linking algorithm.

In view of the charged NH_3_^+^ present on the cross-linking agent, a substitute monomer was used for calculations due to the problems that will occur when periodic boundaries are used for charged systems. The substitute monomer does not change the cross-linking procedure and has no effect on the final configuration of the polymer as the monomer on which NH_3_^+^ resides is a curing agent and will be removed at the end of the curing reaction; refer to Figure 3a for the curing reaction. The details of the substitute cross-linking agent are provided in supporting information.

The above uncrosslinked model contains a total number of 11,040 atoms, with only 8 m-APB molecules and 224 BPh molecules, to satisfy the ratio of 28:1. This model is further equilibrated through Molecular Dynamics (MD) method in order to construct the crosslinked structure in the next step. The equilibration is performed using LAMMPS molecular dynamics software [14]. Consistent Valence Force Field (CVFF) is used in all simulations [15,16]. The non-bonded van der Waals interactions are modelled as the 12-6 Lennard Jones (LJ) potential. The initial structure was formed in a 50 × 50 × 50 Å simulation box with 3D periodic conditions and equilibrated using two MM minimization, one canonical ensemble (NVT) and one isothermal-isobaric (NPT) simulation at 300 K and atmospheric pressure for 100 ps. This is in order to minimize internal forces resulting from the construction of bonds, bond angles and bond dihedrals [12]. After obtaining the equilibrated structure for the uncrosslinked model, the following cross-linking procedure is introduced to construct the final model, which starts with assumptions needed in the procedure, followed by a flow chart of the algorithm and saturation.

### 2.2. Cross-Linking Procedure

The cross-linking reaction of the phthalonitrile system involves the potential formation of a polytriazine, polyphthalocyanine or polyisoindolenine [17]. In our current study, we only considered the reaction pathway involving the formation of polytriazine; refer to Figure 3 for a schematic of the steps leading up to a triazine formation.

The equilibrated structure was statically crosslinked based on the distance between the N atom of the NH_3_^+^ group (NH_2_ in the substituted m-APB) in the activated m-APB cross-linking agent or molecule subsequently (after reaction with BPh monomer) and the C atom of the C≡N group in the BPh monomer. While the actual cross-linking reaction is quite complex, the fundamental mechanisms of the reaction that were modelled in this work are depicted in Figure 3a, in accordance with the cross-linking mechanism established by Burchill [17] where monomers would crosslink via addition polymerization in the presence of a catalyst and undergo trimerization via a condensation reaction forming a triazine. In order to simplify the cross-linking process without losing any important structural information pertaining to the crosslinked structure at each step of the process, the following assumptions were made:

(1) All activated m-APB were assumed to have the same reactivity.
(2) All NH_3_^+^ reactive groups (NH_2_ in substitute m-APB) were assumed to have the same reactivity no matter where their position is on the crosslinked monomer.
(3) A reaction will take place as long as at least one pair of reactive species are within the cutoff distance.
(4) Triazines will form instantaneously at any point in the cross-linking reaction once conditions for its formation are met.

A flowchart describing the cross-linking algorithm is shown in Figure 3b and is discussed below.

Step 1: System is equilibrated using NPT simulations at 600 K (to imitate the curing temperature) for 40 ps. It is important to ascertain that the simulation time in between successive cross-linking reactions is long enough for unreacted species to move around. This simulation time was chosen based on the calculated root-mean-square displacement (RMSD). The RMSD of reaction carbon and nitrogen atoms were found to be ~4.6 and 4.2 Å in 40 ps respectively [12], thus ensuring that the equilibrium time was long enough for various molecules to mix.

Step 2: Initial cutoff distances are defined for the cross-linking algorithm. If and only if reactive species exist within the cutoff distance, the cross-linking reaction will proceed. Atom pairs with minimum distances were selected to create new crosslinked topology due to the relationship between interatomic potential energy and interatomic distance. Reactive atoms (that are at distances ≥ *r_m_*, where *r_m_* is the distance at which the potential reaches its minimum) experience greater attraction with smaller distances which would in turn increase their chances of forming bonds. Thus, the cross-linking methodology was based on having reactive pairs with the minimum distance forming bonds.

Step 3a: If cross-linking does occur, the topological information is updated by introducing new bonds into the system. The system will be scanned for potential structures that are able to form triazines and if those structures exist, the triazine structure will be formed and the activated cross-linking agent will be released back into the system. Thereafter, the topology is relaxed via MM minimization.

Step 3b: If cross-linking does not occur, the system is further equilibrated according to Step 1.

Step 4: After minimization, if cross-linking limit is not reached, the algorithm jumps to Step 1 where the equilibration of the new structure is performed, otherwise, simulations are stopped.

In order to study the properties of the polymer system with respect to cross-linking density, we first define the cross-linking limit as the total number of crosslinks formed in the polymer system when the cutoff distance is set to the space diagonal of simulation box. The crosslink % for other cutoff distances where cutoff distance <space diagonal of simulation box was then defined to be the ratio of the total number of crosslinks that were formed to the cross-linking limit (maximum number of crosslinks possible for that system). Saturation is defined to occur when Loop 1 loops for three consecutive times, at which point, no more cross-linking can take place without extreme perturbation of the system and cross-linking is considered complete.

Once a crosslink is created, charge distribution around the reacted atoms will be altered. In order to account for that, charges were evaluated on a model molecule built with Insight II [15] which has a molecular topology similar to the newly formed cross-link. The original charge distribution was then replaced with the appropriate charges in order to keep the system neutral. The details of the charge redistribution are provided in supporting information.

In order to achieve a relaxed network, the crosslinked system is further equilibrated with one MM minimization, heating at 600 K with NVT equilibration, compression at 600 K and 9000 atm external pressure using NPT equilibration followed by quenching with NPT dynamics to 300 K and 1 atm external pressure. Representative cross-link densities that represent the expected range for a stoichiometric monomer/cross-linking agent ratio were then chosen for subsequent modeling steps.

## 3. Results and Discussion

**Figure 4:**
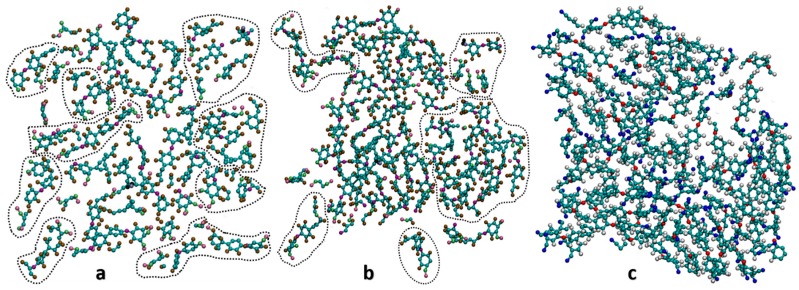
Cross sections of crosslinked phthalonitrile system with (**a**) 25%, (**b**) 50% and (**c**) 100% cross-linking. In (**a**,**b**), different clusters of polymers that are un-crosslinked are circled by dash lines.

Figure 4 shows the cross sections of the final equilibrated structures (for clarity, only in-plane molecules are depicted). Monomers that are already crosslinked are grouped together. Comparing the cross section of 25%, 50% and 100% crosslinked structure, we found that: (a) The number of circled clusters decreases as the cross-linking density increases, which means monomers are increasingly crosslinked together, from totally uncrosslinked (depicted in Figure 2) to 100% crosslinked (depicted in Figure 4c) which is the formation of one big molecule from all the linked monomers; (b) the density of the system increases as cross-linking level increases because monomers become closer to each other. The volume of the system shrinks gradually during the process of network formation, as observed in experiments. For highly dense system (100% cross-linking), a volume shrinkage of about 7% (relative to its volume at 0% cross-linking) is observed in our simulations. This is comparable with 12.8% reported by Keller [18] for phthalonitrile resin heated at 500 °C for 24 h.

### 3.1. Thermal Properties

**Figure 5:**
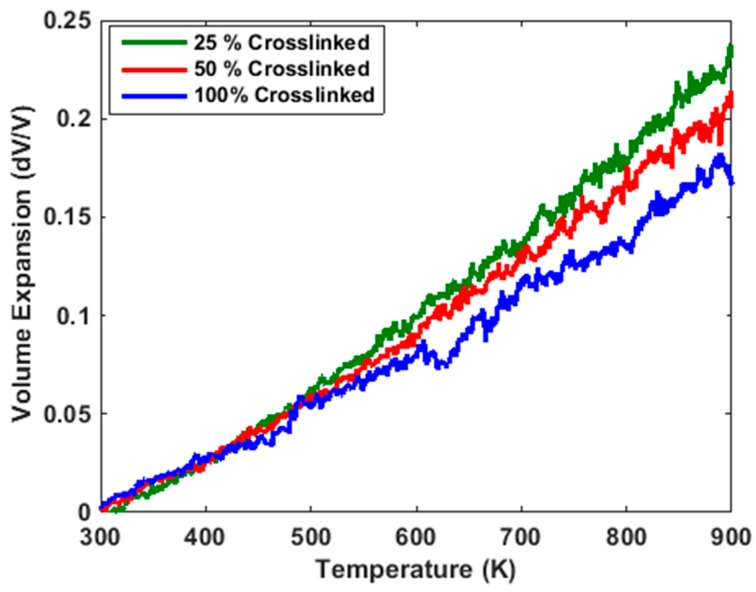
Volume expansion with respect to temperature for 25%, 50% and 100% crosslinked structures.

Thermal expansion coefficients were used to study the thermal property of this material. Volume change dV with respect to the initial volume *V*_0_ (at 300 K) for the 25%, 50% and 100% crosslinked systems in the temperature range 300–900 K were determined and plotted in the Figure 5 below. Each crosslinked system was equilibrated at 1 atm for 30 ps before an NPT (constant temperature and constant pressure) simulation was conducted where the polymer was slowly heated from 300 K to 900 K. From the plot, it is clear that the 50% crosslinked polymer system exhibited a significantly larger volume expansion than the 100% crosslinked system. This is due to the reduced number of covalent bonds in systems with lower cross-linking densities, which will allow more monomers to move around freely.

Linear regression lines were fitted on the volume expansion curves in Figure 5 to determine the coefficient of volumetric thermal expansion (CVTE) in both the rubbery and glassy regimes. In the glassy regime, the data were fitted for the 300 K–450 K temperature range and for the rubbery regime, the data were fitted for 750 K–850 K. Given that the *T*_g_ of phthalonitrile polymer systems is around 670 K–700 K, the coefficient of linear thermal expansion (CLTE) was then calculated using a method similar to that used by Ananyo et al. [10].

The CLTE values of the crosslinked systems are shown in Table 1 and generally indicate an increase in the coefficient of expansion with decreasing crosslink density which is expected due to increased mobility of molecule chains at lower crosslink densities. The results were found to be consistent with those reported by Itecma [19] which reported an experimental CLTE value of 9.0 × 10^−5^ K^−1^ when the PN resin was postcured to 180 °C and a value of 3.5 × 10^−5^ K^−1^ when the resin was postcured to 375 °C.

### 3.2. Mechanical Properties

Simulated compression tests were performed on 25% crosslinked, 50% crosslinked and 100% crosslinked systems to determine their elastic properties. In these simulations, uniaxial compressive strain and uniaxial tensile strain with a strain rate of 10^12^/s were imposed on the MD models with periodic boundary conditions in the NPT ensemble. NPT simulations were run at 300 K for 10 ps. Strain increments were applied at every timestep such that the desired cumulative strain was reached by the end of the simulation. The elastic responses of polymers are generally dependent on applied strain rate [20,21] (with the young’s modulus increasing by up to 4 times in the case of PMMA when strain rate was varied from ~10^−3^/s to ~10^3^/s) and it is to be noted that for the current MD simulations, the strain rate used is very much higher than experimental strain rates which are typically on the order of 10^−4^/s. As a result, the strain rate hardening which results from a much higher strain rate explains the discrepancy between experimental specific tensile strengths of ~36 MPa·cm^3^/g [19] compared to the computed specific compressive strength shown in the graph below of ~1000 MPa·cm^3^/g.

**Figure 6:**
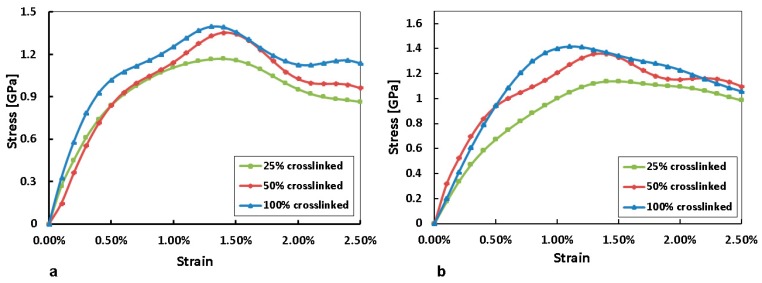
Graph of stress versus strain at different crosslink % (**a**) tensile strength; (**b**) compressive strength.

By comparing the results of the system at different cross-linking percentages in Figure 6 below, it is apparent that the yield stress of the 100% crosslinked system is the highest followed by 50% crosslinked and 25% crosslinked. The percentage strain at which the maximum yield stress occurred is noted to decrease with increasing crosslink percent. This is in agreement with the typical behavior of polymeric systems, with more highly crosslinked systems having a greater load bearing ability than systems that are not as highly crosslinked [22]. The higher strain percentage at which systems with lower levels of crosslink yield is also in accordance with polymeric behavior where the greater the molecular weight of the molecule chains, the stiffer the polymer will be, resulting in brittle behavior with yield occurring at low strain percentages [23]. This trend is reflected in the Young’s modulus by calculating the slope of the linear part of curves in Figure 6b, with values of 143.4 GPa, 174.0 GPa and 201 GPa, for 25%, 50% and 100% crosslinked PN respectively. The increasing tendency of Young’s modulus is indicative of higher stiffness for higher crosslink densities.

### 3.3. Dielectric Properties

We proceed with the calculation of the static dielectric constant using the different crosslinked systems. Fix E-field command in LAMMPS was used in the molecular dynamics simulation for an empirical approximation to how charged particles react in the presence of an external E-field. For the polymeric system under uniaxial displacement, the dielectric constant can be defined in Equation (1).

(1) $$k_{11}=k_{0}\left(1+\chi_{11}\right)$$

$k_{11}$ is the dielectric constant along axial direction, $k_{0}=8.854\times 10^{-12}\;F/m$ is the vacuum permittivity, $\chi_{11}$ is the susceptibility of the medium, which is a function of axial polarization density $P_{1}$ and the E-field applied to the material:

(2) $$\chi_{11}=\;\frac{1}{k_{0}}\frac{\partial P_{1}}{\partial E_{1}}$$

The axial polarization $P_{1}$ is determined from $P_{1}=P_{1}^{e}+P_{1}^{d}$, where $P_{1}^{e}$ is the polarization due to the relative displacement of electrons and core and $P_{1}^{d}$ denotes the polarization due to the relative displacement between atoms. Following the molecular dynamics simulation done by Zhang [24] the effect of polarization between the nucleus and electron cloud is neglected in this study, which results in elimination of the clamped ion term i.e., $P_{1}^{e}=0$. The axial polarization vector thus can be written as:

(3) $$P_{1}=P_{1}^{d}=\;\sum_{i=1}^{N}\frac{x_{1}^{i}q_{i}}{\bar{V}}$$

where $q_{i}$ and $x_{1}^{i}$ are, respectively, the electric charge and the coordinate along the axial direction of atom *i*, *N* is the number of atoms and $\bar{V}$ is the volume of the polymer mass.

To calculate dielectric constant, different E-field values are applied across one axis of the PN model, the structure is relaxed and the coordinates of all its atoms in the final relaxed state is recorded. The polarization densities under different *E* values are obtained according to Equation (3). A $P_{1}$−$E_{1}$ curve is obtained and the slope equals to the electrical susceptibility $\chi_{11}$ defined in Equation (2). A detailed derivation and steps to get $\chi_{11}$ are shown in supporting information.

**Figure 7:**
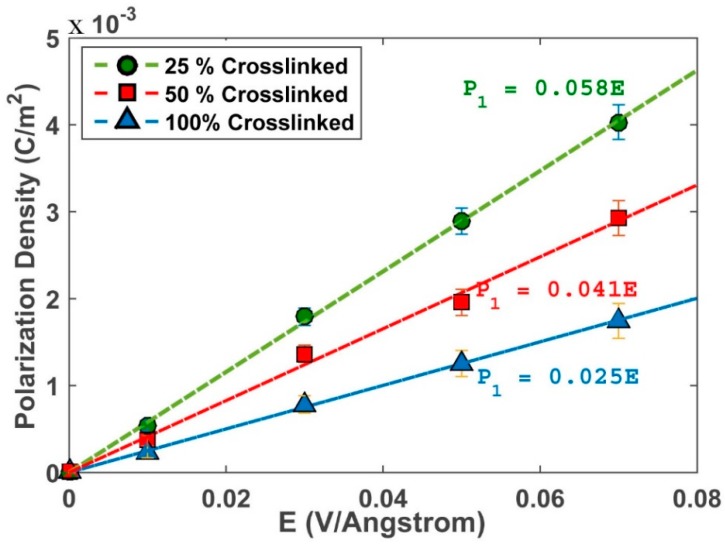
Graph of normalized polarization density versus electric field for different crosslinked systems.

The $P_{1}$−$E_{1}$ curves of different crosslinked systems are plotted in Figure 7.

**Figure 8:**
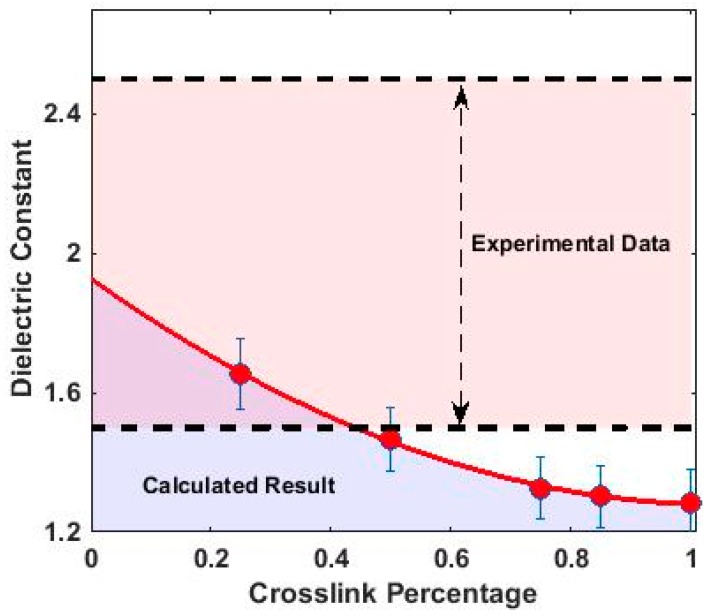
Graph of dielectric constant versus % crosslink.

As expected, a linear relation between electric field and polarization density is found, whose slope is proportional to the susceptibility of the system $\chi_{11}$; this value decreases as the cross-linking level increases, because the material becomes rigid and less polarizable upon the imposition of an electric field. Converting $\chi_{11}$ to dielectric constant through Equation (1), we get the final dielectric constants of different systems, as shown in Figure 8.

Dielectric constants calculated above are indicative of an insulating material. The value decreases as the level of crosslink density increases and an empirical quadratic function $D=0.57\rho^{2}-1.22\rho +1.92$ can be used to fit the relation between dielectric constant (D) and cross-linking density (ρ). A comparison was conducted between calculated values and experimental data of similar PN systems [4,25,26] and intersections between them were found for lower crosslink densities.

As shown in Figure 8, the calculated dielectric constant is relatively low compared to the experimental range. This is probably due to the symmetry of triazine structures modelled in this work as well as the decrease in mobility of structures with higher crosslink densities. Under laboratory conditions, it is difficult to control the trimerization of crosslinked monomers. Not every crosslinked monomer will form a triazine structure and the polymeric system will likely have triazine and linear chains of crosslinked monomers existing together. The polarizability of the system will increase with the increase of asymmetrical linear chains due to a net polarization which explains the increasing difference between experimental results and modelled results as cross-linking percentage increases. Additionally, a 100% crosslinked polymer structure hinders orientational polarization due to its decreased molecular mobility. The polymer mixes that were used to obtain the experimental dielectric constant were unlikely to be 100% crosslinked systems, contributing to increased orientational polarizability of molecule chains, leading to a higher measured dielectric constant. As such, the calculated dielectric constant for a 100% crosslinked polymer system provides a useful upper limit reference for the above modelled phthalonitrile polymer system.

## 4. Conclusions

The present article provides a detailed and clear discussion on the molecular modelling study of a crosslinked BPh–m-APB phthalonitrile polymeric system modelled to crosslink densities that are difficult to attain experimentally (100% crosslinked). This material shows excellent mechanical property with high strength and low dielectric constant.

We also found that the level of crosslink density is a very important factor affecting the material’s properties. As the cross-linking density increases, the material shows better resistance to mechanical deformation, and becomes a better insulator with a lower dielectric constant. We also studied the limiting case of 100% cross-linking via computational methods, which shows the best mechanical and electric properties; in this aspect, we are able to extend the understanding of this material, overcoming experimental limitations of synthesizing a 100% crosslinked polymer.

Although the procedure is discussed for BPh–m-APB phthalonitrile system, the same approach can be used for other crosslinked networks. Simulations were carried out to investigate the coefficient of thermal expansion of the phthalonitrile model and both structural as well as electrical properties were investigated as a function of crosslink %. Both were found to agree well with typical polymeric behavior and experimental results, a noteworthy achievement showing the potential of using MD tools for molecular simulation of polymers. These cross-linking algorithms are currently being used for the study of the carbonization process of phthalonitrile polymeric systems. Studies are also undertaken to optimize for the smallest simulation box size that is still representative of the properties of the PN polymeric system in order to investigate more material properties while saving on computational resources.

## Figures and Tables

**Table 1:** Coefficients of linear thermal expansion under different cross-linking densities, in unit 10^−5^ K^−1^.

CLTE	25%	50%	100%
Above *T*_g_	15.13	13.70	12.86
Below *T*_g_	9.99	8.77	6.99

## Supplementary Materials

The following are available online at www.mdpi.com/2073-4360/10/1/64/s1.
