# Supplementary material for: A Molecular Dynamics Study of Crosslinked Phthalonitrile Polymers: The Effect of Crosslink Density on Thermomechanical and Dielectric Properties
Source: Polymers (Basel). 2018 Jan 11;10(1):64. doi: 10.3390/polym10010064 (PMC6414959; doi:10.3390/polym10010064)
Supplement: Supplementary file 1 [file polymers-10-00064-s001.pdf]

Article

A Molecular Dynamics Study of Crosslinked Phthalonitrile Polymers: The Effect of Crosslink Density on Thermomechanical and Dielectric Properties

Janel Chua [1] and Qingsong Tu [2,*]

[1] DSO National Laboratories, Singapore 118225, Singapore; csonglin@dso.org.sg
[2] Department of Civil and Environmental Engineering, University of California, Berkeley, CA 94720, USA
* Correspondence: howietu@berkeley.edu

Table S1. Elemental Composition Analysis.

Composition	Carbon%	Hydrogen%	Nitrogen%
PN resin	72.46	3.24	15.45
PN foam	70.78	3.24	14.29

Elemental analyses of the specimens were conducted using Elementar's vario MICRO cube CHNS analyzer. The samples were broken up with a mallet and the required amount (3 mg) was collected for analysis. The CHNS analyzer in NUS is not equipped to detect oxygen which is present in the PN resin. As such, percentages of C, H and N do not add up to 100%.

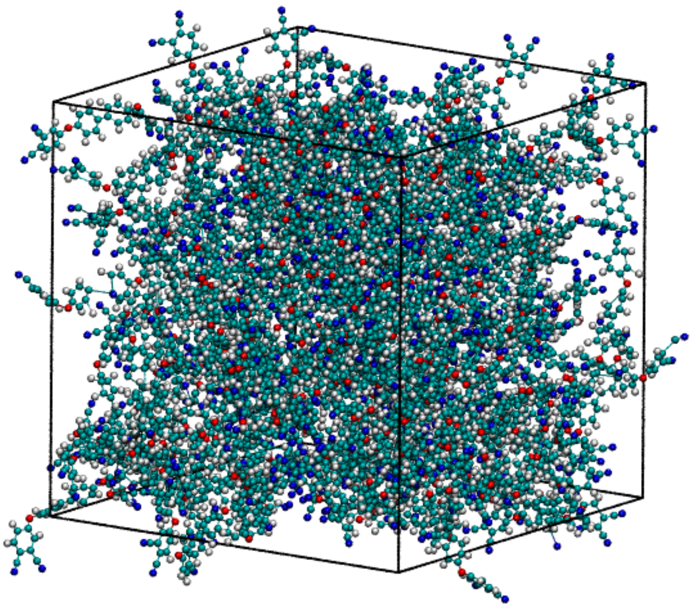

Figure S1. Initial uncrosslinked model of BPh – m-APB of ratio (28:1) containing 11,040 atoms.

Substituting charged curing agent with non-charged curing agent

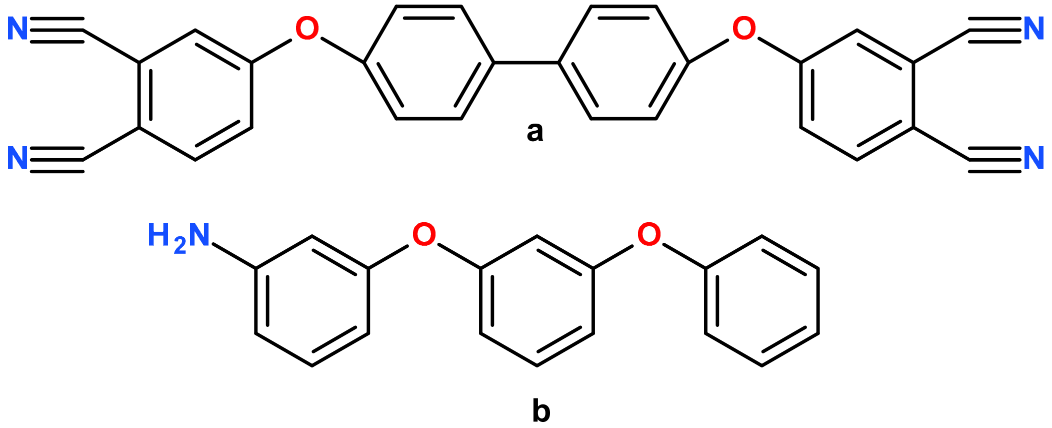

Figure S2. Molecular structure of a) BPh monomer and b) modified m-APB cross-linking agent used as a substitute for the charged m-APB cross-linking agent.

The substitute cross-linking agent replaces the NH_3^+ reactive site with NH_2. The cross-linking procedure conducted with BPh monomer and this substituted m-APB cross-linking agent is otherwise the same. This substitution prevents potential problems that may arise when periodic boundary conditions are imposed on charged systems, exponentially increasing even the smallest of net charges into a large net charge that results in an unstable system.

Saturation

Figure S3 to Figure S5 below provide an illustration of the charge redistribution that occurred at various stages of crosslink.

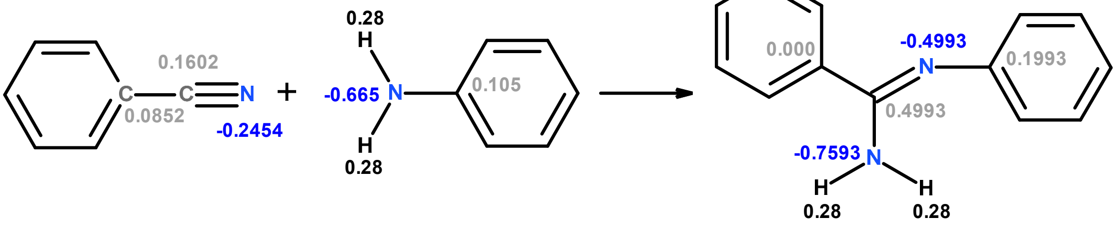

Figure S3. Case 1 when the modified m-APB curing agent first links to a BPh monomer.

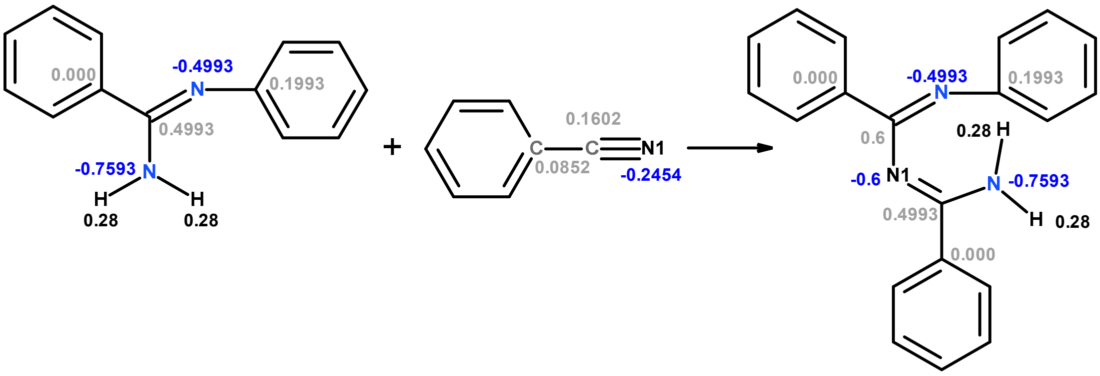

Figure S4. Case 2 subsequent addition cross-linking steps where other BPh monomers bind to a BPh monomer that was previously reacted with a modified m-APB curing agent.

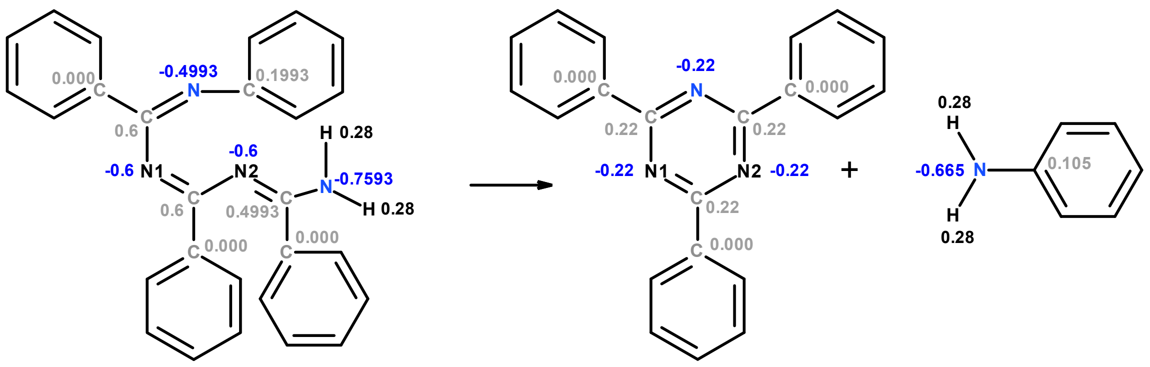

Figure S5. Case 3 formation of Triazine and release of the modified curing agent.

Dielectric Constant Calculations

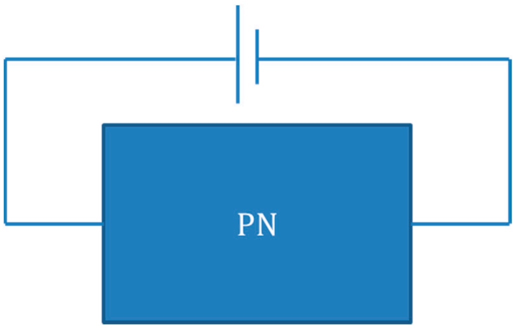

Figure S6. Schematic showing the application of E-field on the 100% crosslinked system.

To calculate the dielectric constant, one must solve the following constitutive relation for the polymeric system

$$D_i = e_{iq}\lambda_q + k_{ik}E_k \tag{S1}$$

where λ_q is the strain tensor, k_{ik} the dielectric constant, e_{iq} is the transpose of the piezoelectric coefficient, D_i and E_k are, respectively, the electric displacement and the electric field.

According to Equation (S1) above and considering axial electric displacement $D_1 = k_0E_1 + P_1$ (where k_0 is the vacuum permittivity and P_1 is the axial polarization), k_{11} can be defined as

$$k_{11} = (\partial D_1/\partial E_1)_{\lambda_q} = k_0(1 + \chi_{11}) \tag{S2}$$

$$\chi_{11} = (\delta P_1/\delta E_1)_{\lambda_q}/k_0\,, \quad k_0(\text{vacuum permittivity}) = 8.854 * 10^{-12}\ F/m \tag{S3}$$

$$P_1(\text{axial polarization density}) = \sum_{i=1}^{N} \frac{x_1^i q_i}{\bar{V}} \tag{S4}$$

The dielectric constant of the material can be expressed as Equation (S2) where χ_{11} is the susceptibility of the medium. χ_{11} is a function of axial polarization density P_1 and the E-field applied to the material. To calculate dielectric constant, an E-field of fixed value is first applied across one axis of the PN model, the structure is relaxed and the coordinates of all its atoms in the final relaxed state is recorded. The polarization density is obtained using Equation (S4), where q_i and x_1^i are, respectively, the electric charge and the coordinate along the axial direction of atom i, N is the number of atoms and $\bar{V}$ is the volume of the polymer mass. Having obtained a few polarization densities for a few E-field values applied across the PN model, a $P_1 - E_1$ curve may be drawn with a slope equal to the electrical susceptibility χ_{11} defined above in Equation (S3). Using Equation (S2) along with the value for χ_{11}, dielectric constant k_{11} can then be calculated.

It is noted that the axial polarization P_1 is determined from $P_1 = P_1^e + P_1^d$, where P_1^e is the polarization due to the relative displacement of electrons and core and P_1^d denotes the polarization

due to the relative displacement between atoms. Following the molecular dynamics simulation done by Zhang[1], the effect of polarization between the nucleus and electron cloud is neglected in this study, which results in elimination of the clamped ion term i.e., $P_1^e = 0$. The axial polarization vector thus can be written as $P_1 = P_1^d = \sum_{i=1}^{N} x_1^i q_i / \bar{V}$.

In order for the P_1 versus E_1 curve to be accurate, it had to be ensured that the system had truly relaxed before using the atomic coordinates for polarization density calculation. As such, the convergence of the polarization density value over relaxation time was monitored and a log shaped curve was observed after 60ps of relaxation which indicates that the system was converging to a P_1 value.

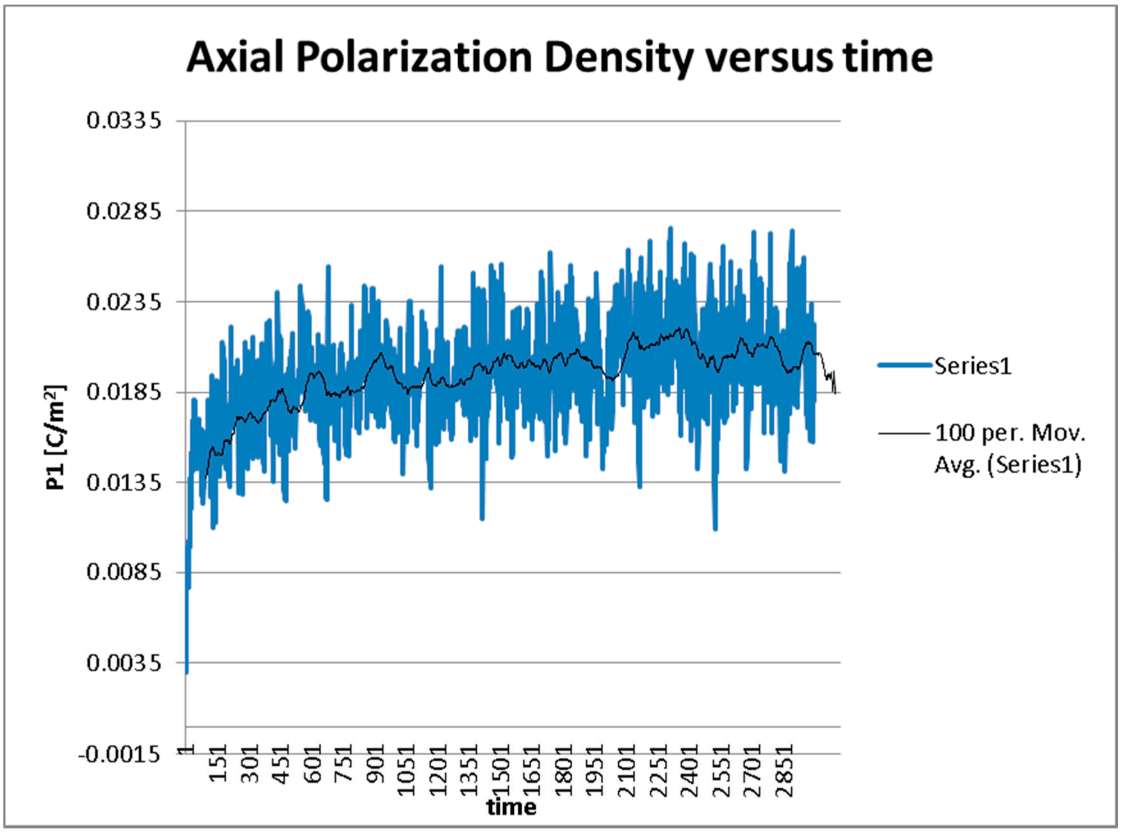

Figure S7. Graph of axial polarization density versus timestep.

A few more P_1 values were obtained for different E-fields and the resulting plot can be seen in the Figure S8 below. According to Equation (S3), the gradient of the line is the susceptibility of the medium χ_{11} .Finally, Equation (S2) was used to calculate the dielectric constant k_{11}.

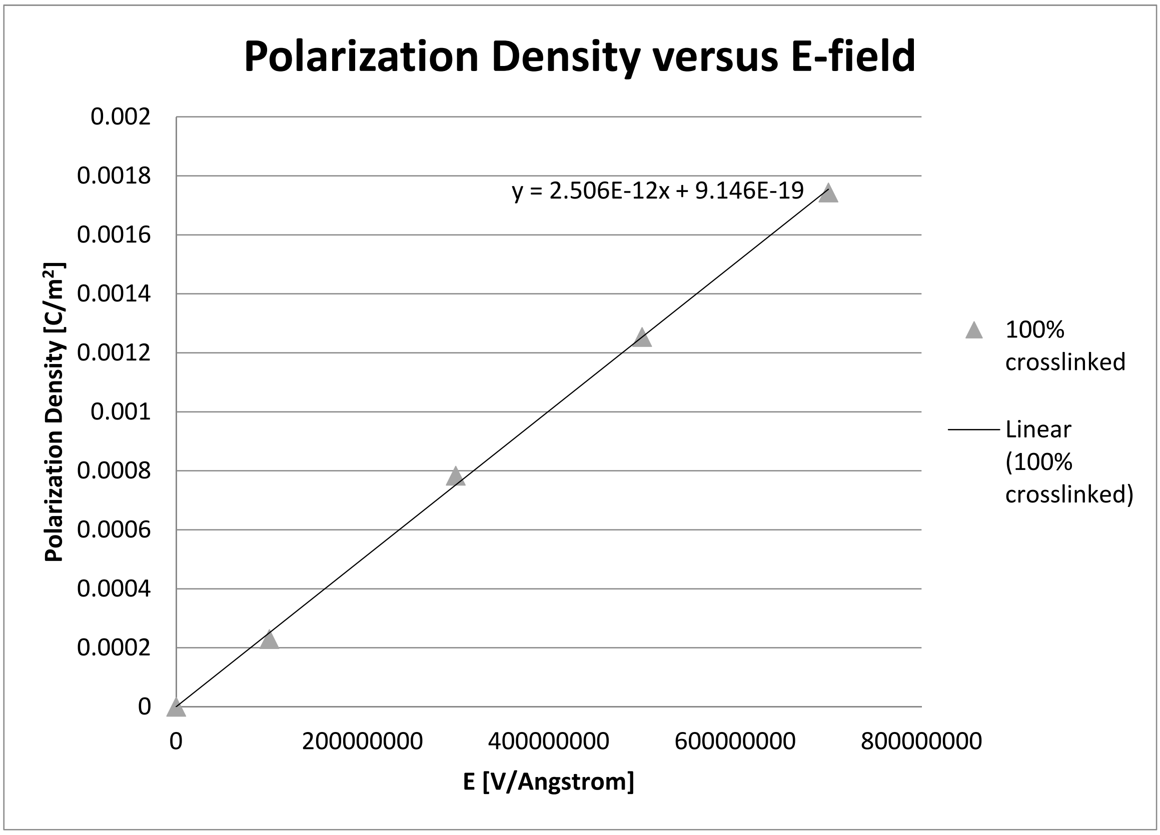

Figure S8. Graph of polarization density versus electric field.

$$\delta P_1/\delta E_1 = 2.506 * 10^{-12}$$

$$\chi_{11} = \frac{\left(\frac{\delta P_1}{\delta E_1}\right)}{k_0} = \frac{2.506 * 10^{-12}}{8.854 * 10^{-12}} = 0.283$$

$$\frac{k_{11}}{k_0} = 1.283, \text{Insulating material}$$

References

1. Zhang, J. Small-scale effect on the piezoelectric potential of gallium nitride nanowires. *Appl. Phys. Lett.* **2014**, *104*, 253110.
